# Computed tomography-detected extramural venous invasion-related gene signature: a potential negative biomarker of immune checkpoint inhibitor treatment in patients with gastric cancer

**DOI:** 10.1186/s12967-022-03845-2

**Published:** 2023-01-05

**Authors:** Hao Yang, Xinyi Gou, Caizhen Feng, Yinli Zhang, Fan Chai, Nan Hong, Yingjiang Ye, Yi Wang, Bo Gao, Jin Cheng

**Affiliations:** 1 Department of General Surgery, The Second Affiliated Hospital of Harbin Medical University, Harbin, China; 2 Department of Radiology, Peking University People’s Hospital, 11 Xizhimen South St., Beijing, 100044 China; 3 Department of Pathology, Peking University People’s Hospital, Beijing, China; 4 Department of Gastrointestinal Surgery, Peking University People’s Hospital, Beijing, China; 5 Department of General Surgery, Peking University People’s Hospital, Beijing, 100044 China

**Keywords:** Radiogenomics, Extramural venous invasion, Immunotherapy, Gastric cancer

## Abstract

**Background:**

To investigate the association between computed tomography (CT)-detected extramural venous invasion (EMVI)-related genes and immunotherapy resistance and immune escape in patients with gastric cancer (GC).

**Methods:**

Thirteen patients with pathologically proven locally advanced GC who had undergone preoperative abdominal contrast-enhanced CT and radical resection surgery were included in this study. Transcriptome sequencing was multidetector performed on the cancerous tissue obtained during surgery, and EMVI-related genes (*P* value for association < 0.001) were selected. A single-sample gene set enrichment analysis algorithm was also used to divide all GC samples (n = 377) in The Cancer Genome Atlas (TCGA) database into high and low EMVI-immune related groups based on immune-related differential genes. Cluster analysis was used to classify EMVI-immune-related genotypes, and survival among patients was validated in TCGA and Gene Expression Omnibus (GEO) cohorts. The EMVI scores were calculated using principal component analysis (PCA), and GC samples were divided into high and low EMVI score groups. Microsatellite instability (MSI) status, tumor mutation burden (TMB), response rate to immune checkpoint inhibitors (ICIs), immune escape were compared between the high and low EMVI score groups. Hub gene of the model in pan-cancer analysis was also performed.

**Results:**

There were 17 EMVI-immune-related genes used for cluster analysis. PCA identified 8 genes (PCH17, SEMA6B, GJA4, CD34, ACVRL1, SOX17, CXCL12, DYSF) that were used to calculate EMVI scores. High EMVI score groups had lower MSI, TMB and response rate of ICIs, status but higher immune escape status. Among the 8 genes used for EMVI scores, CXCL12 and SOX17 were at the core of the protein–protein interaction (PPI) network and had a higher priority in pan-cancer analysis. Immunohistochemical analysis showed that the expression of CXCL12 and SOX17 was significantly higher in CT-detected EMVI-positive samples than in EMVI-negative samples (*P* < 0.0001).

**Conclusion:**

A CT-detected EMVI gene signature could be a potential negative biomarker for ICIs treatment, as the signature is negatively correlated with TMB, and MSI, resulting in poorer prognosis.

## Introduction

Gastric cancer (GC) is the fifth most common cancer and the fourth-leading cause of cancer-associated death globally [1]. Multiple treatments are used to improve survival in patients with GC, and immune checkpoint inhibitors (ICIs) have recently emerged as the most advanced therapeutic option available for patients with advanced GC [2]. The discovery of novel biomarkers associated with the tumor immune reaction may allow for innovative approaches in patient selection for immunotherapy, potentially leading to improved treatment response. For instance, previous studies have shown that several biomarkers, including microsatellite instability (MSI), programmed death-ligand 1 (PD-L1) expression, tumor mutation burden (TMB), Epstein-Barr virus (EBV) status, and Pold/Pole, may be associated with a response to anti-programmed death receptor 1(PD-1)therapy [3, 4], and recent research has suggested that MSI high status could be a predictive biomarker for the efficacy of ICIs treatment in patients with GC [5, 6]. However, information about these biomarkers was obtained via pathological specimens. Because of the high heterogeneity of GC, the site from which the specimen is taken can greatly affect the information obtained about biomarkers [7]. In contrast to biopsy, imaging studies can provide a more comprehensive view of each case of GC. Despite this, no previous studies have assessed the potential connection between gross imaging features and response to ICI therapy.

Extramural venous invasion (EMVI) has been identified as another potential biomarker in advanced GC. Pathologically, EMVI is defined as tumor cells actively invading the venous lumen beyond the muscularis propria [8]. EMVI therefore plays a critical role in distant metastatic development. Multiple studies have indicated that EMVI can be effectively diagnosed as a gross imaging feature on computed tomography (CT) in patients with GC [9]. Furthermore, CT-detected EMVI is considered an independent predictor of distant metastases in patients with GC [9–11]. Because EMVI is closely associated with tumorous vessels, blood vascular endothelial cells play essential roles in controlling the microenvironment and cross-modulating the immune response [12]. In previous work, we were able to establish an EMVI-prognosis-related model that could predict overall survival in patients with GC [13]. Where the seven gene signatures included ENPEP, which is known to be associated with immune responses. Based on this information, we hypothesize that the development of EMVI is not only generated by the tumor cell itself but is also mediated by the tumor microenvironment and may be associated with immunotherapy.

Using the principles of radiogenomics [14], we therefore aimed in this study to investigate the relationship between EMVI-related genes and immune cell infiltration, tumor mutation burden, microsatellite instability, and long-term survival. Furthermore, we sought to investigate the association between CT-detected EMVI and immunotherapy resistance and immune escape.

## Materials and methods

### Research design

The institutional review board approved this retrospective analysis (approval number: 2019PHB171-01) and waived the requirement for informed consent. This study included 13 patients with locally advanced GC (pathologically proven tumor [T], node [N], and metastatic [M] status of T4aN + M0 as defined by the American Joint Committee on Cancer [AJCC]). All patients underwent contrast-enhanced multidetector CT (ceMDCT) scans, followed by curative surgery, chemotherapy, and follow-up. The frozen cancerous samples were all stored in the institute biobank.

**Fig. 1:**
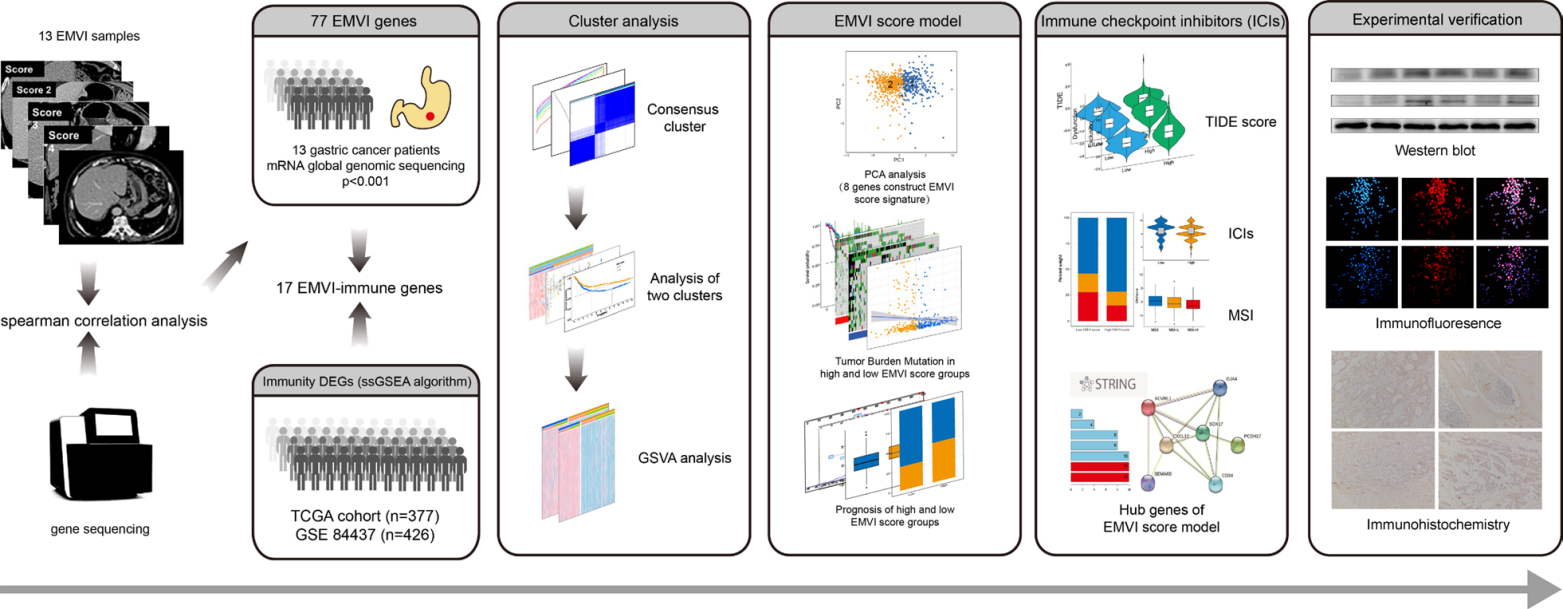
Flowchart of the study

The flowchart of the study is shown in Fig. 1. First, the study patients underwent preoperative abdominal ceMDCT. Tissue samples were assessed with transcriptome sequencing, and EMVI-related genes that demonstrated *P* values < 0.001 on Spearman analysis were selected for further analysis. The immunity scores for all GC patients in The Cancer Genome Atlas (TCGA) were then calculated using a single-sample gene set enrichment analysis (ssGSEA) algorithm, and patients were divided into high- and low- immunity groups based on these scores. The EMVI-immune-related genes were then generated by overlapping the immunity score differential genes (DEGs) and EMVI-related genes. Cluster analysis using the "ConsensusClusterPlus" package in R was then used to classify all GC samples in TCGA according to EMVI-immune-related genes. Next, the immune cell infiltration status of GC clusters was determined using the ssGSEA algorithm and the gene ontology (GO) and Kyoto Encyclopedia of Gene and Genomes (KEGG) analyses. The EMVI-immune-related gene scores for all GC samples in TCGA were then calculated using principal component analysis (PCA). The parameter lambda was adjusted using cross-validation, and the final EMVI-immune-related genes were selected to construct an EMVI score model. Next, all GC samples were classified into high and low EMVI score groups. Sensitivity to ICIs and immune evasion were also analyzed. A protein–protein interaction (PPI) network and modules of EMVI gene signature were established, and related pan-cancer analysis was performed using the "reshape2" and "ggpubr" packages. Finally, we performed western blotting and immunofluorescence of the core in PPI on GC cells and immunohistochemistry staining on formalin-fixed paraffin-embedded (FFPE) tissues from the patients initially included in the study.

### CT-detected EMVI scoring and mRNA sequencing

CT-detected EMVI of GC could be scored from 0 to 4 [15], with scores of 0 to 2 defined as EMVI negative and scores of 3 or 4 defined as EMVI positive. Of the 13 included patients, 2 had EMVI scores of 1, 5 had scores of 2, 5 had scores of 3, and 3 had scores of 4. No patients had a score of 0, as all patients had T4a disease, meaning that the masses had a nodular appearance.

After quality control testing, frozen tumor tissue samples from these 13 patients underwent transcriptome sequencing using the Illumina HiSeq 4000 system. We used the R language to calculate the Spearman correlation between gene expression and EMVI score, with the threshold of correlation significance set at *P* < 0.001. A heat map was used to illustrate EMVI-related genes and the clinical data. The sequencing data had been uploaded to the Gene Expression Omnibus (GEO) database (GSE182831).

### Gene data collection and processing

Through TCGA (https://portal.gdc.cancer.gov, accessed December 2021), we obtained raw data for the mRNA matrix in fragments per kilobase million (FPKM) format for GC, as well as the copy number data. The mRNA matrix raw data were processed to remove duplicate samples. We also obtained clinical data related to GC patients from TCGA. Through the GEO database (accessed December 2021), we downloaded the GSE84437 dataset and obtained the mRNA matrix and clinical data for GC patients. The FPKM matrix was converted into transcript per million (TPM) format and merged with the GEO matrix, and some missing genes were removed by batch correction. GO and KEGG files were downloaded from the GSEA website (https://www.gsea-msigdb.org, accessed December 2021). We then downloaded the Tumor Immune Dysfunction and Exclusion (TIDE) score, exclusion score, and dysfunction data from the TIDE database (http://tide.dfci.harvard.edu, accessed December 2021), as well as the GC's immune checkpoint therapy score data from The Cancer Immunome Atlas (TCIA) (https://tcia.at, accessed December, 2021) and the pan-cancer mRNA matrix raw and gene mutation data from the UCSC database (xena.ucsc.edu/December, 2021). A PPI network was constructed using the STRING website (https://string-db.org/cgi/input.pl, accessed December 2021).

### ssGSEA

Through the ssGSEA algorithm, we calculated the immune cell infiltration status and immunity scores for all GC samples in TCGA. We divided the samples into high and low immune groups based on these immunity scores. The Estimate of Stromal and Immune cells in Malignant Tumors (ESTIMATE) was used to calculate the stromal score, immune score, and estimate score for the high and low immune groups. An immune microenvironment heat map was then drawn for the high and low immune groups. Using differential analysis (false discovery rate [FDR] < 0.05), we identified DEGs in the high and low immune groups.

### Cluster analysis of EMVI-immune-related genes

We classified GC data using the "ConsensusClusterPlus" R package to verify the association between EMVI-immune-related genes and GC. A range of 2 to 9 was used; a κ value equaling 2 was considered the ideal number of clusters, as the correlation between groups was weak whereas the correlation within groups was strong. Using the ssGSEA algorithm, we determined the expression of immune cells in different GC clusters and illustrated them with boxplots. GO and KEGG analyses were performed for EMVI-immune-related genes. The "GSEABase" package and the R language "GSVA" package were then used to create a heat map of enriched functional pathways in GC clusters.

### EMVI-immune-related gene signature construction

PCA was performed using the stats R package to calculate EMVI scores and to identify EMVI-immune-related genes for all GC patients. The penalty parameter (λ) was determined using tenfold cross-validation, and patients were then stratified into high and low EMVI score groups based on the median value of the EMVI score. The association between EMVI score and TMB was calculated using the "survival" and "survminer" R packages. The correlation between EMVI score and immune cell infiltration was analyzed using the ssGSEA algorithm. Correlations between EMVI score and clinical-pathological characteristics were illustrated with histograms and box plots using the "plyr" and "ggpubr" P packages. A NOMO model was constructed to classify risk factors as predictors of survival.

### Response to ICIs treatment

We calculated the TIDE score, exclusion score, and dysfunction score to identify immune escape and immune dysfunction status in the high and low EMVI score groups. Using the EMVI score, we also analyzed the cytotoxic T-lymphocyte-associated antigen 4 (CTLA-4) and PD-1 immune checkpoints therapy in GC. MSI status was analyzed in the high and low score groups. A PPI network for EMVI-immune-related genes was constructed, and the core proteins and hub-genes were identified.

### Cell culture

GC cells (GES-1, AGS, HGC-27, KATO III, MKN-1, MKN-45) were purchased from Procell Life Science & Technology (Wuhan, China), and the cells were cultured according to the manufacturer’s instructions. The cell lines were cultured in RPMI-1640 medium (Gibco, USA) and were supplemented with 10% fetal bovine serum (FBS) (Gibco) and 1% penicillin/streptomycin (Gibco).

### Western blotting for GC cells

Western blotting was performed as described previously [16]. In brief, we used radioimmunoprecipitation assay (RIPA) buffer to extract the proteins needed for western blotting. Sodium dodecyl sulfate–polyacrylamide gel electrophoresis was then performed to separate the proteins, which were transferred to poly (vinylidene fluoride) membranes. The following antibodies were used: anti-SOX17 (Abcam, USA), anti-SDF1/CXCL12 (Abcam), and GAPDH (ImmunoWay, USA). Corresponding Alexa Fluor dyes were used for fluorescent detection. DAPI was used for nuclear counter staining. Images were captured on a Zeiss LSM780 laser scanning confocal microscope.

### Immunohistochemistry on FFPE GC tissues

FFPE cancerous tissues from the 13 study patients were cut into 5-μm sections. Immunohistochemical (IHC) staining was performed using anti-SOX17 and anti-CXCL12. Tissue sections were deparaffinized in xylene and rehydrated in graded ethanol. Antigen retrieval was performed by heating sections in boiling sodium citrate buffer (Sigma-Aldrich, C-9999) for 20 min. After blocking was performed using 3% hydrogen peroxide and bovine serum albumin (BSA), the tissues were incubated with the primary antibody at 4 °C overnight. After being washed, the tissues were incubated with corresponding horseradish peroxidase (HRP)-conjugated secondary antibodies. Color was developed using diaminobenzidine (DAB) substrate (Sigma-Aldrich, D-7304), and slides were counterstained with hematoxylin. Images of three random areas from each section were captured at 20× magnification.

### Pan-cancer analysis

The “reshape2” and “ggpubr” packages were used to draw a sorted boxplot of single gene expression in pan-cancer. Single-gene TMBs in pan-cancer samples were calculated, and radar charts of TMB and MSI were drawn using the "fmsb" package. With the "CIBERSORT" algorithm, the expression of immune cells in pan-cancer samples was calculated, and a co-expression heat map of pan-cancer immune cells was drawn.

### Statistical analysis

The copy number variation frequencies of EMVI-related genes were obtained by calculating the gene copy number gains and deletions in GC samples from TCGA. The number of mutations in genes was calculated and waterfall charts were drawn. Using the "RCircos" package, a gene copy number circle diagram was drawn. Cox regression and co-expression analyses were used to construct a prognostic network associated with EMVI-related genes. Kaplan–Meier curves and log-rank analyses were used to compare survival among GC patients, and plots were constructed using the "survminer" package. A nomogram and receiver operating characteristic (ROC) curve were then constructed for risk stratification based on EMVI score and other clinical-pathological characteristics in GC patients.

## Results

### Clinical data and EMVI-related gene selection

CT-detected EMVI and clinical characteristics of the 13 study patients are shown in Table 1. A total of 77 EMVI-related genes demonstrated *P* values < 0.001.

**Table 1:** Baseline characteristics of patients from TCGA and GEO database

Clinical feature	Total patients (n = 803)		TCGA (n = 377)		GSE21501 (n = 426)	
	Number	%	Number	%	Number	%
Age						
≤ 65 y	456	56.79	176	46.68	280	65.73
> 65 y	347	43.21	201	53.32	146	34.27
Sex						
Female	265	33.00	130	34.48	135	31.69
Male	538	67.00	247	65.52	291	68.31
Follow-up status						
Alive	451	56.16	227	60.21	224	55.58
Dead	352	43.84	150	39.79	202	47.42
Grade						
1–2	135	16.81	135	35.81	0	0
3	242	30.14	242	64.19	0	0
Unknown	426	53.05	0	0	426	100
Stage T						
1–2	147	18.31	98	25.99	49	11.50
3–4	656	81.69	279	74.01	377	88.50
Stage N						
N0	196	24.41	116	30.77	80	18.78
N1–3	607	75.59	261	69.23	346	81.22

### Genotyping of GC according to immune score

**Fig. 2:**
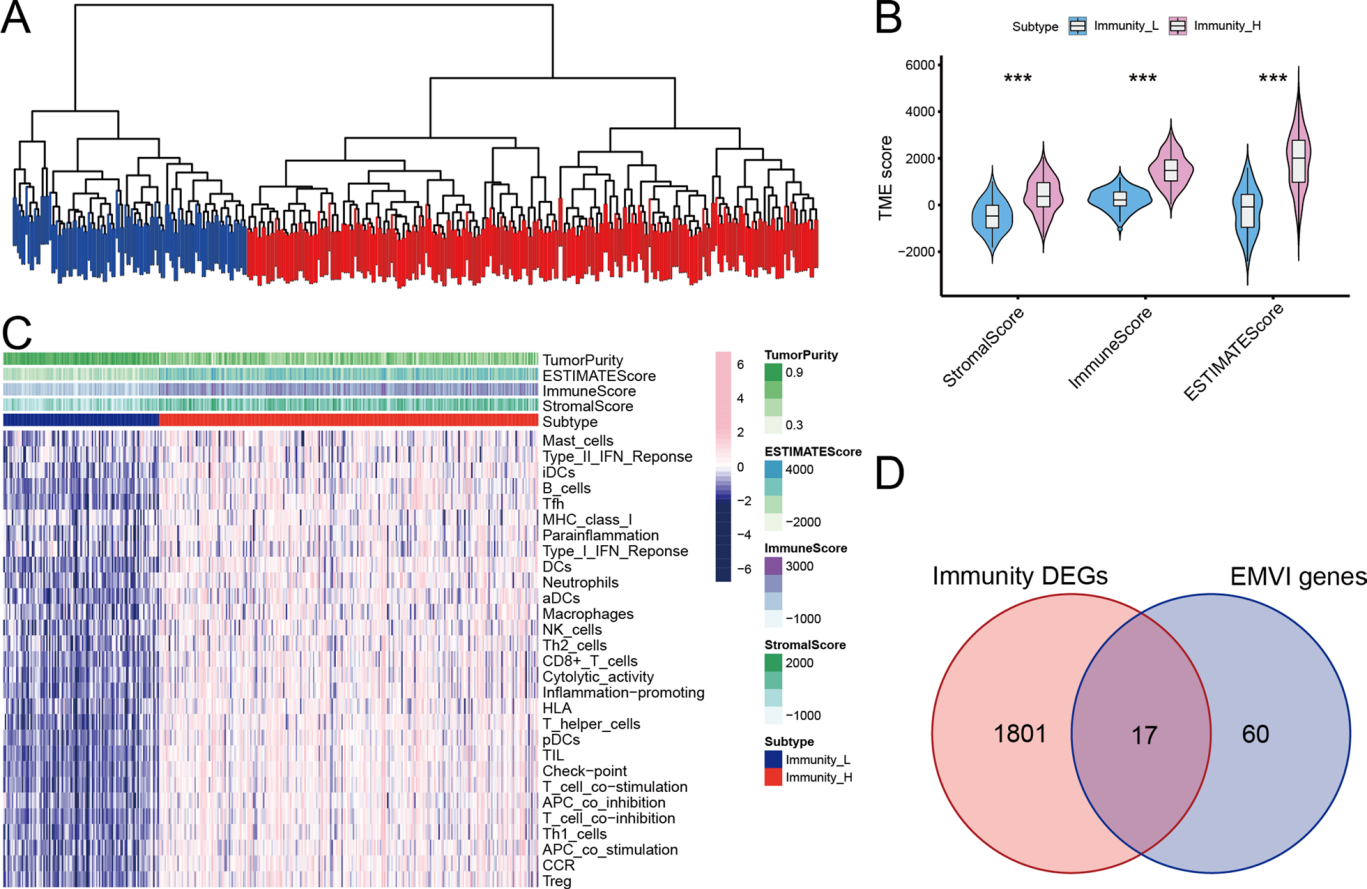
Subtyping of GC based on immunity score. **A** 377 GC patients in TCGA were subtyped using the ssGSEA algorithm and divided into high and low immunity groups. **B** Stromal, immune, and ESTIMATE scores were significantly increased in the high immunity group. **C** An immune microenvironment heatmap demonstrated a significant difference in immune cell infiltration between the two groups. **D** Seventeen EMVI-immune-related genes were identified by overlapping immune-related DEGs and EMVI-related genes. ***P < 0.001

All GC samples (n = 377) in TCGA were divided into high and low immune groups (Fig. 2A). The stromal score, immune score, and estimate score were significantly higher in the high immunity group than in the low immunity group (Fig. 2B). The immune microenvironment heatmap also demonstrated a significant difference in immune cell infiltration between the groups (Fig. 2C). Differential analysis identified 1818 immune-related DEGs; when the immune-related DEGs were overlapped with EMVI-related genes, a total of 17 EMVI-immune-related genes were identified (Fig. 2D).

### Expression of EMVI-immune-related genes in GC

**Fig. 3:**
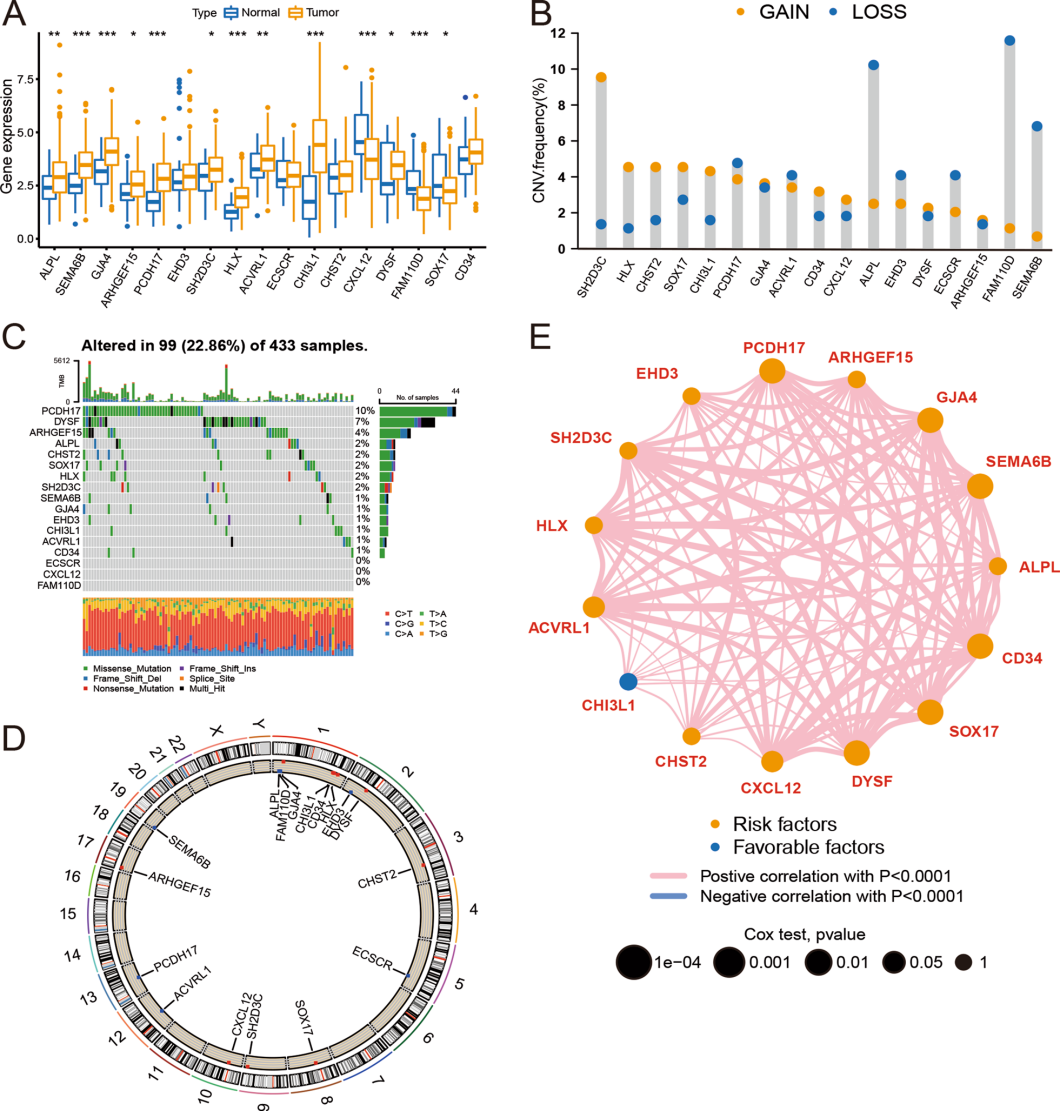
Expression characteristics of EMVI-immune-related genes. **A** The expression of EMVI-immune-related genes was significantly higher in GC tissue than in normal gastric tissue. **B** Diagram of the frequency of copy number variations in EMVI-immune genes. **C** Single-gene mutation frequency waterfall plot. (D) Gene copy number circle plot. **E** Prognostic network diagram through co-expression analysis showed that most EMVI-immune-related genes were risk factors. *P < 0.05; **P < 0.01; ***P < 0.001

The expression of most EMVI-immune-related genes differed significantly between normal gastric tissue and GC tissue (Fig. 3A). The most obvious copy number gain was SH2DSC, and the most obvious copy number loss was FAM110D (Fig. 3B). For EMVI-immune-related genes, the single-gene mutation frequency was 22.86%, among which PCDH17 (10%) and DYSF (7%) had the highest mutation rates (Fig. 3C, D). Among the EMVI-related genes, only CHI3L1 was a favorable predictor of prognosis; the remaining genes were risk factors (Fig. 3E).

### Cluster analysis of EMVI-immune-related genes

**Fig. 4:**
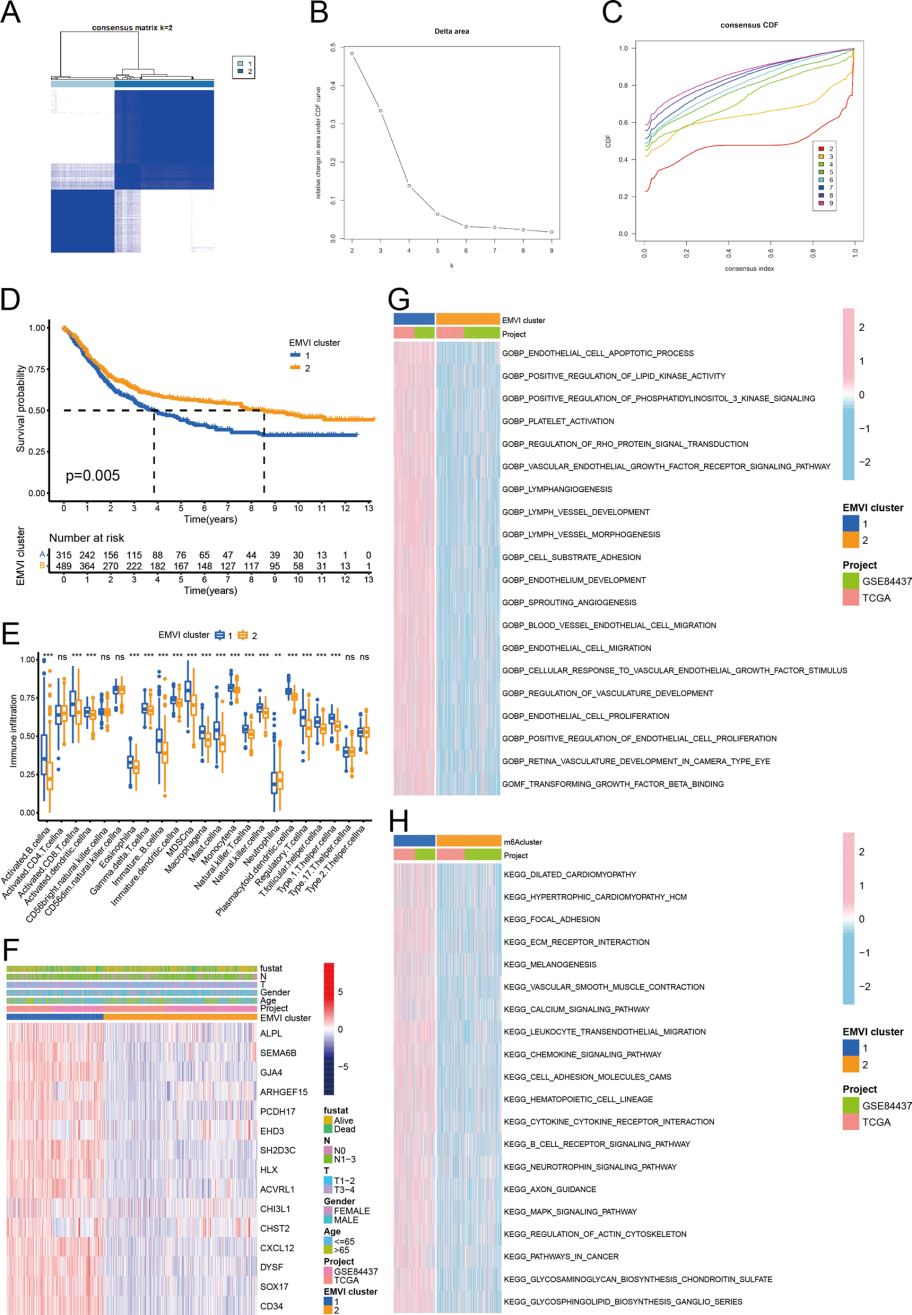
Cluster analysis of EMVI-immune genes. **A**–**C** Cluster analysis of EMVI -immune genes with GC samples divided into two clusters. **D**, **E** Cluster 1 showed decreased overall survival and increased immune cells. **F** Heatmap of the clinical-pathological features of GC samples and the EMVI-immune genes. **G**, **H** GO and KEGG analyses of the EMVI-immune-related genes. **P < 0.01; ***P < 0.001; ns: no significance

All GC samples were divided into two clusters (Fig. 4A–C). Survival analysis demonstrated that the survival rate of cluster 1 was significantly lower than that of cluster 2 (Fig. 4D). We calculated the expression of 23 immune cells in the two clusters of GC and found that 18 types demonstrated significant differences in expression (Fig. 3E). A heatmap was created to demonstrate the clinical-pathological features of GC samples and EMVI-immune genes (Fig. 4F). Further heatmap analysis demonstrated that GO enrichment pathways in cluster 1 were mainly concentrated in the areas of cell proliferation and endothelial cell metastasis (Fig. 4G), such as endothelial cell migration and endothelial cell proliferation. In cluster 1, KEGG-enriched pathways were mainly concentrated in the tumor microenvironment and oncogenic pathways (Fig. 4H), such as ECM (extracellular matrix) receptor interaction, focal adhesion, and pathways in cancer.

### EMVI score generated by PCA

**Fig. 5:**
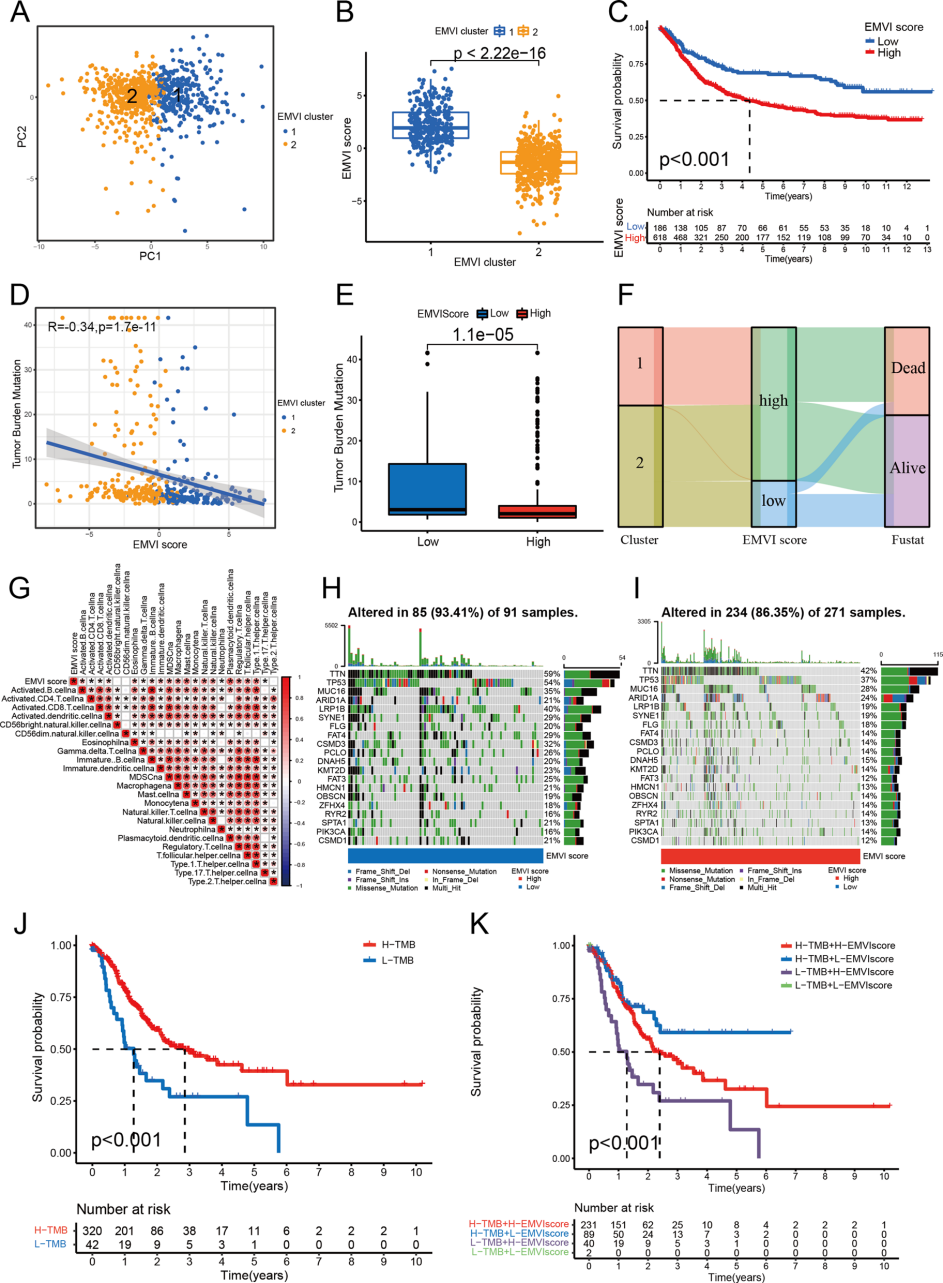
EMVI score generated by PCA. **A**, **B** High and low EMVI score groups were generated by PCA. **C** The survival rate of the high EMVI score group was significantly lower than that of the low EMVI score group. **D**, **E** EMVI score was negatively correlated with TMB status. **F** Sankey diagram of EMVI immune gene cluster, EMVI score, and patient survival. **G** Multiple immune cells were related to EMVI score. **H**, **I** The single-gene mutation frequency in the low EMVI score group was higher than that in the high EMVI score group. **J** The survival rate of the high TMB group was significantly higher than that of the low TMB group. **K** Combined analysis of the high and low TMB groups and the high and low EMVI score groups. *P < 0.05

**Fig. 6:**
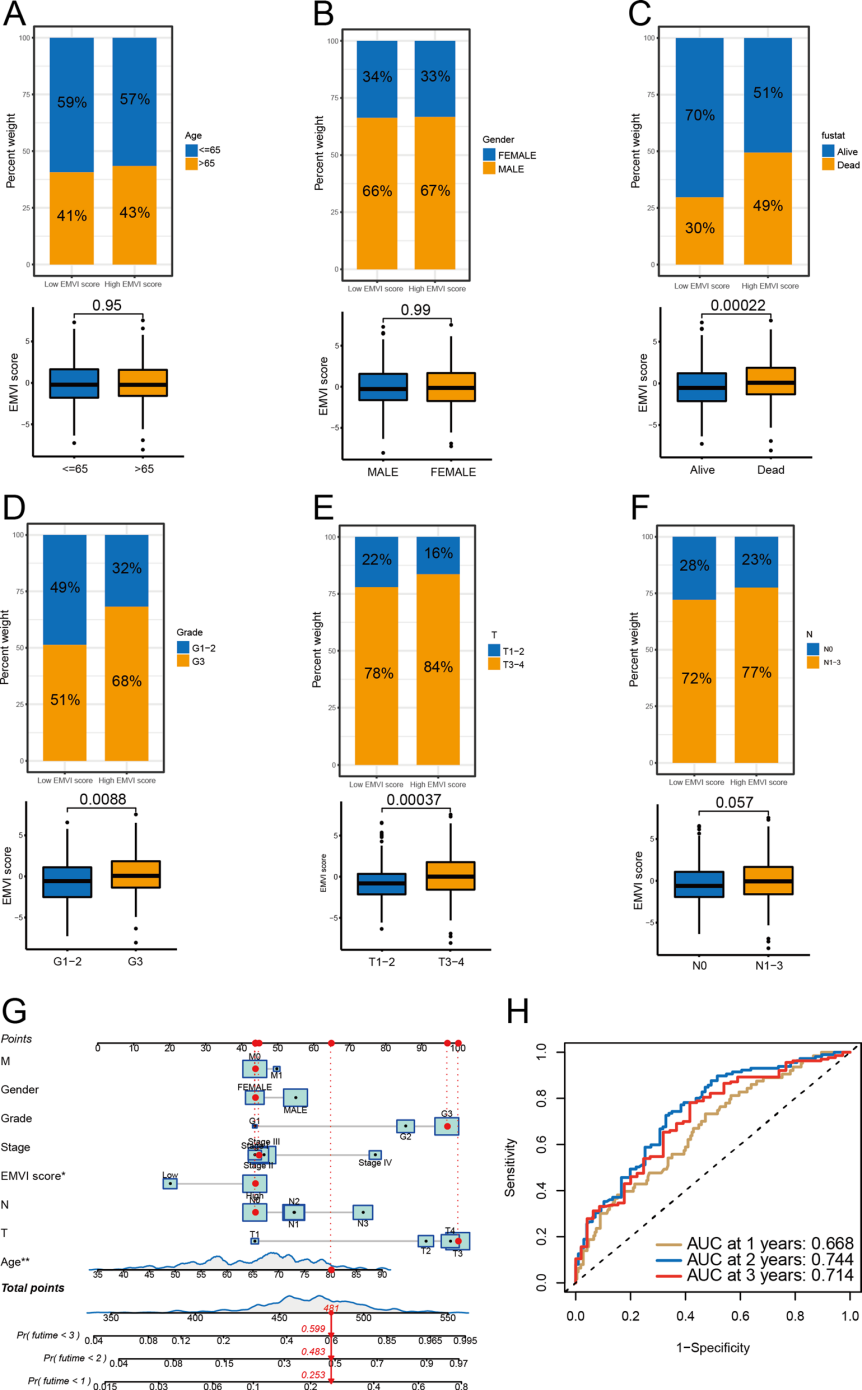
Prognostic analysis of high and low EMVI score groups. **A**, **B** Age and sex of the patients in the high and low EMVI score groups were not statistically different. **C**–**F** There were significant differences between high and low EMVI score groups in prognosis, tumor differentiation grade, and T stage, but not N stage. **G** NOMO model demonstrated that if the patient's total score reached 481, the 1-, 2-, and 3-year mortality rates were 25.3%, 48.3%, and 59.9%, respectively. **H** The AUC values at 1, 2, and 3 years were all greater than 0.65

Through PCA of all GC samples (Fig. 5A), we found that there was basically no overlap between the two clusters, and there was a good correlation within the cluster. Eight genes (PCH17, SEMA6B, GJA4, CD34, ACVRL1, SOX17, CXCL12, DYSF) were selected to construct the EMVI score model, and GC samples were divided into high and low EMVI score groups. The EMVI score in cluster 1 was significantly higher than that in cluster 2 (Fig. 5B), and the survival rate of the high EMVI score group was significantly lower than that of the low EMVI score group (Fig. 5C). EMVI score was also found to be negatively correlated with TMB status (Fig. 5D, E). A Sankey diagram demonstrated that samples from cluster 1 were all divided into the high EMVI score group, whereas samples of the low EMVI score group were divided into cluster 2. Most cases of patients who had died came from the high EMVI score group, whereas the vast majority of cases in the low EMVI score group were patients who had survived. (Fig. 5F). Correlation analysis between the EMVI score and immune cell infiltration (Fig. 5G) showed that 20 of the 23 immune cells were correlated with the EMVI score. The frequency of single-gene mutations was higher in the low EMVI score group than in the high EMVI score group (Fig. 5H, I), whereas the survival rate was significantly higher in the high TMB group than in the low TMB group (Fig. 5J). A combined analysis of different TMB and EMVI score showed that patients with high TMB and low-EMVI scores would earn favourite survival. On the contrary, low TMB with a high-EMVI score corresponded to poor survival (Fig. 5K). There were significant differences in survival, tumor grade, and T stage between the different EMVI score groups (Fig. 6). A NOMO model demonstrated that if the patient's total score reached 481, the 1-, 2-, and 3-year mortality rates were 25.3%, 48.3%, and 59.9%, respectively (Fig. 6G). The area under the curve (AUC) values at 1, 2, and 3 years were all greater than 0.65 (Fig. 6H).

### Response to ICIs treatment

**Fig. 7:**
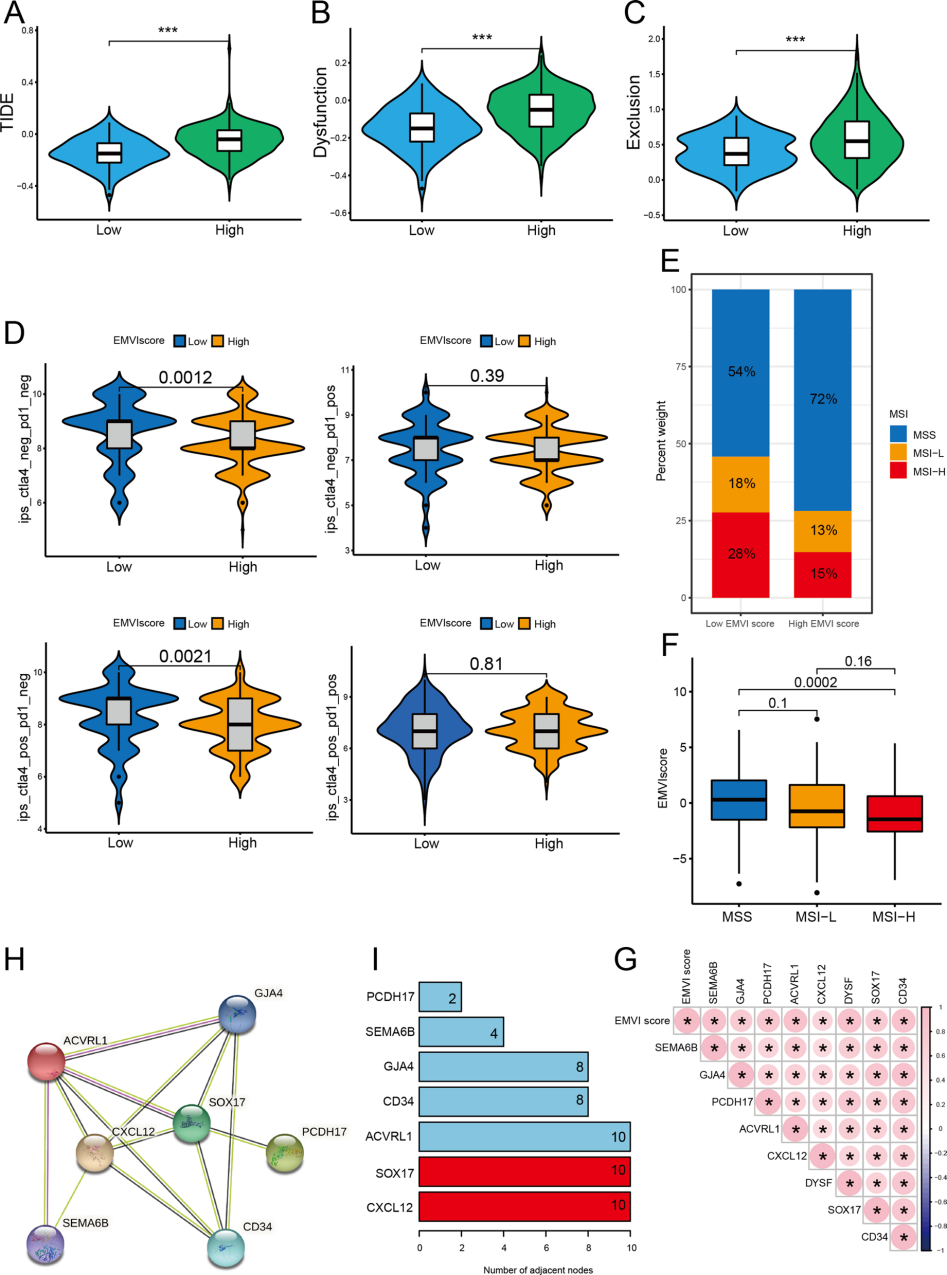
ICIs, immune escape, and PPI network. **A**–**C** TIDE score, exclusion score, and dysfunction score were significantly higher for the high EMVI score group than for the low EMVI score group. **D** Patients with a high EMVI score had a lower response rate to ICIs therapy (PDL1). **E**, **F** The low EMVI score group had higher microsatellite instability (MSI). **G** Through using correlation analysis, we found that EMVI scores were positively correlated with 8 EMVI proteins. **H**, **I** PPI network for EMVI-related genes showed that CXCL12 and SOX17 were at the core of the network. MSS: microsatellite stable group, L-MSI: low MSI group; H-MSI: high MSI group. *P < 0.05; *** *P* < 0.001

The TIDE score, exclusion score, and dysfunction score of the high EMVI score group were significantly higher than those of the low EMVI score group (Fig. 7A–C). EMVI score and ICIs treatment effect were closely related; patients with a high EMVI score had a lower response rate to ICI therapy (PDL1) (Fig. 7D). Furthermore, the low EMVI score group had higher MSI status (Fig. 7E, F). Correlation analysis demonstrated that EMVI scores were positively correlated with 8 EMVI proteins (Fig. 7G), and the EMVI proteins were able to interact to form a functional ensemble. Analysis of the PPI network for EMVI-related genes demonstrated that CXCL12 and SOX17 were at the core of the network (Fig. 7H, I).

### Expression analysis of EMVI-related hub genes CXCL12 and SOX17

**Fig. 8:**
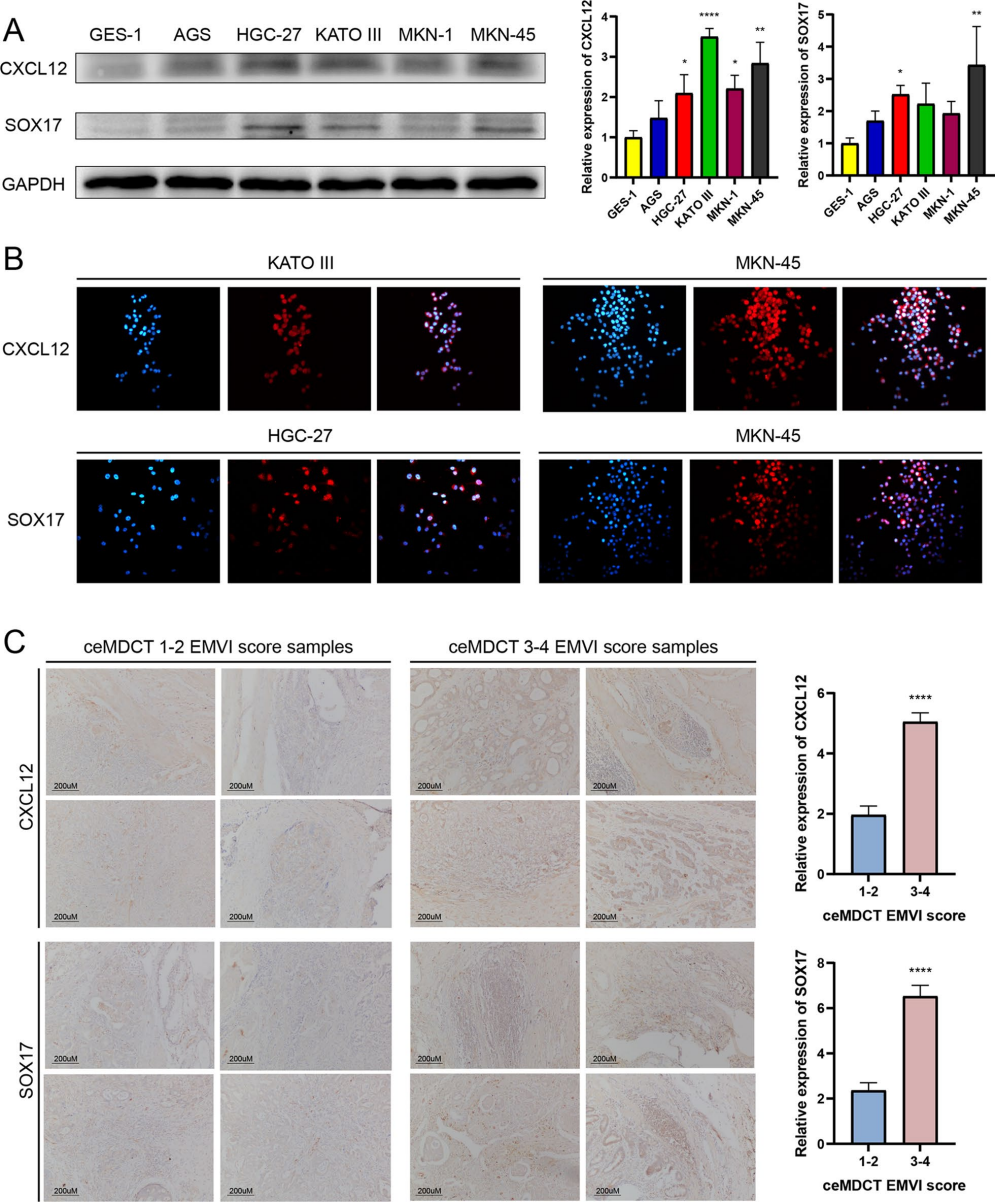
Expression analysis of CXCL12 and SOX17 in GC cell lines and clinical samples. **A** Western blot showed that CXCL12 had the highest expression in KATO III and MKN-45 cell lines, and SOX17 had the highest expression in HGC-27 and MKN-45 cell lines. **B** The results of immunofluorescence analysis were consistent with the results of western blot. **C** IHC analysis showed that the expression of CXCL12 and SOX17 in CT-detected EMVI-positive samples was significantly higher than that in CT-detected EMVI-negative samples. *P < 0.05; **P < 0.01; ***P < 0.001; ****P < 0.0001

CXCL12 had the highest expression in KATO III (*P* < 0.0001) and MKN-45 (*P* < 0.001) cell lines. SOX17 had the highest expression in HGC-27 (*P* < 0.05) and MKN-45 (*P* < 0.01) cell lines (Fig. 8A). The results of immunofluorescence were consistent with the results of western blot (Fig. 8B). IHC demonstrated that the expression of CXCL12 and SOX17 in CT-detected EMVI-positive samples was significantly higher than in CT-detected EMVI-negative samples (*P* < 0.0001) (Fig. 8C).

### Pan-cancer analysis of EMVI-related hub genes

**Fig. 9:**
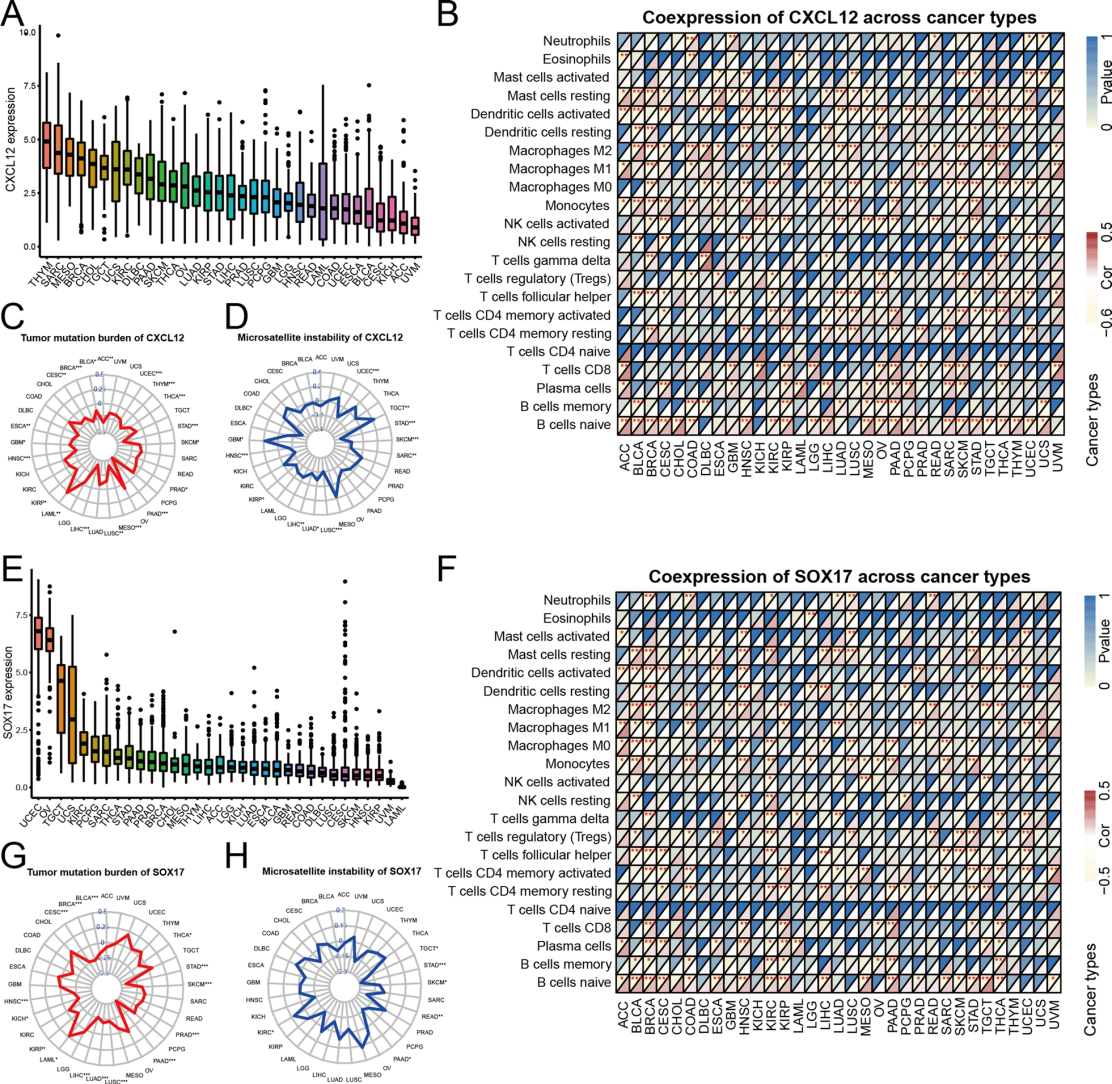
Analysis of CXCL12 and SOX17 in pan-cancer. **A**, **E** Expression of CXCL12 and SOX17 in pan-cancer. **B**, **F** The two genes were closely related to immune cell infiltration in various tumors. **C**, **D**, **G**, **H** Radar maps for TMB and MSI of CXCL12 and SOX17 in pan-cancer. A cor value < 0 indicated that the gene was negatively correlated with TMB and MSI. CXCL12 and SOX17 were negatively correlated with TMB and MSI in various tumors. *P < 0.05; **P < 0.01; ***P < 0.001; ****P < 0.0001

Figure 9A and E demonstrate the expression of CXCL12 and SOX17 in pan-cancer, respectively. Both genes were relatively highly expressed in GC, and highly expressed in multiple gastrointestinal tumors, including cholangiocarcinoma (CHOL), pancreatic adenocarcinoma (PAAD), and liver hepatocellular carcinoma (LIHC). These two genes were closely related to immune cell infiltration in various tumors (Fig. 9B, F), and CXCL12 and SOX17 were negatively correlated with TMB and MSI in various tumors (Fig. 9C, D, G, H).

## Discussion

The main finding of this study was that the PCA-defined EMVI score gene model was negatively correlated with TMB, MSI, and overall survival. Furthermore, the TIDE score, exclusion score, and dysfunction score in the high EMVI score group were significantly higher than those in the low EMVI group. Immune escape led to lower response rate fo GC patients to ICIs therapy. In addition, the genes CXC12 and SOX17 were found to be at the core of the PPI network.

Recently, ICIs have emerged as among the most advanced therapeutic options available for patients with advanced GC [2]. The FDA approved pembrolizumab in May 2017 for patients with unresectable or metastatic MSI-H or mismatch repair deficient (dMMR) solid tumors that progressed even after prior treatment and for those patients with no other optimal treatment choices [17]. The current study found that the proportion of MSI-H in the high EMVI score group was significantly lower than in the low EMVI score group, which explains the poor response to immunotherapy and poorer long-term prognosis in the high EMVI score group. Proteins made from gene mutations can be recognized as tumor antigens. Mutations can quickly occur during the gene replication process of cell division due to the genetic instability of tumor cells [18]. Neoantigens produced by genetic mutations can induce a strong tumor immune response [19]. This explains why TMB was found to be an indicator of immunotherapy in this study, with the high EMVI score group demonstrating decreased TMB and single gene mutations.

Previous studies have shown that immune escape is essential for tumor survival and development and can potentially lead to immunotherapy resistance [20]. In this study, the high EMVI score group showed significantly higher TIDE, exclusion, and dysfunction scores than the low EMVI score group. This finding suggests that the high EMVI score group is more prone to immune dysfunction and immune rejection than immune escape leading to immunotherapy resistance. We also found that EMVI score and immune checkpoint treatment effect were closely related, as patients with high EMVI scores had a lower response rate to an ICI. This suggests that patients with high EMVI scores are more likely to be resistant to PD1 immunotherapy.

The presence of various immunosuppressive factors in the tumor microenvironment poses a formidable barrier to T-cell infiltration and function [21]. The tumor microenvironment contains a network of immunosuppressive factors capable of inhibiting T-cell function despite the activated immune response against the tumor achieved through immunotherapy [22]. Within the 8-gene EMVI score model, CXC12 was identified as the core gene in the PPI network. Previous studies have reported that CXC12 plays an essential role in immune evasion by recruiting infiltrating Myeloid-derived suppressor cells (MDSCs) into the tumor microenvironment [23, 24]. In this study, EMVI immune-related cluster 1 had elevated immune scores but decreased overall survival, potentially because of immune escape. In addition, the elevated levels of immune cells included not only CD8+ but also MDSCs cluster 1. However, the detailed diagnostic methods and cutoffs for MDSCs are diverse and not yet standardized [25]. Tumor infiltrating lymphocytes (TILs) have also been studied as potential prognostic markers and therapeutic targets in recent years. Neoantigens are presented on cancer cells, leading to recruitment of TILs and triggering an immune reaction. This could explain why TIL was not identified as an indicator of immunotherapy effectiveness in this study. Although higher TIL density has repeatedly been reported as a favorable prognostic biomarker, its diagnostic method is again not standardized, and there is no consensus regarding the cutoff for high TIL density.

The EMVI score signature in this study included 8 genes: PCH17, SEMA6B, GJA4, CD34, ACVRL1, SOX17, and CXCL12. In addition to CXC12, SOX17 was also at the core of the PPI network. The SOX family is correlated with multiple clusters of immune cells and is involved in the regulation of the angiogenesis-related signaling pathway [26]. In one study, SOX17 was found to promote high-grade glioma by increasing vascular endothelial growth factor (VEGF) mediated vascular abnormality [27]. EMVI is defined as the invasion of a tumor into the extramural veins; thus, EMVI may be associated with VEGF-mediated angiogenesis. Furthermore, CD34 and activin-A receptor cluster II-like kinase 1 (ACVRL1) have also been associated with angiogenesis [28]. In addition, ACVRLI has been shown to regulate transforming growth factor (TGF)-beta, which is an immunosuppressive cytokine that plays an essential regulatory role in the tumor microenvironment [29]. Based on how the EMVI score gene signature functions, the mechanism of CT-detected EMVI may be associated with immunology and angiogenesis, which accords with the immune-vascular cross-talk mechanism [30]. SEMA6B has also been shown to correlate with tumor invasion and metastasis in GC [31] and to be closely related to the immunosuppressive microenvironment in colorectal cancer [32].

In this study, there were significant differences in prognosis, tumor grade, and T stage between the high and low EMVI score groups, similar to findings from previous research [33]. However, there was no significant difference between low and high EMVI score groups in N stage. That may be due to the offset of clinical data from the patients, still indicating that high EMVI scores represent a higher degree of malignancy in terms of clinical-pathological characteristics.

This study had two main limitations. First, EMVI-related genes were generated from 13 GC samples. However, for the aim of gene mining, 6:7 is generally considered enough. Second, this was a bioinformatics study; in vitro and in vivo verification is still needed.

In conclusion, based on the principles of radiogenomics, we established a gene model for CT-detected EMVI. This model was found to be negatively correlated with immune cell infiltration, TMB, MSI, immune escape, and immunotherapy resistance, suggesting that the model could potentially be used as a negative biomarker for ICIs treatment in patients with GC.

## Data Availability

The original contributions presented in the study are publicly available. The data can be found in the GEO database (accession number GSE182831).

## Abbreviations

CT: Computed tomography
EMVI: Extramural venous invasion
GC: Gastric cancer
TCGA: The Cancer Genome Atlas
GEO: Gene Expression Omnibus
PCA: Principal component analysis
MSI: Microsatellite instability
TMB: Tumor mutation burden
ICIs: Immune checkpoint inhibitors
PPI: Protein–protein interaction
PD-L1: Programmed death-ligand 1
EBV: Epstein-Barr virus
PD-1: Anti-programmed death receptor 1
ceMDCT: Contrast-enhanced multidetector CT
ssGSEA: Single-sample gene set enrichment analysis
DEGs: Differential genes
GO: Gene ontology
KEGG: Kyoto Encyclopedia of Gene and Genomes
FFPE: Formalin-fixed paraffin-embedded
FPKM: Fragments per kilobase million
TPM: Transcript per million
TIDE: Tumor Immune Dysfunction and Exclusion
TCIA: The Cancer Immunome Atlas
ESTIMATE: Estimate of Stromal and Immune cells in Malignant Tumors
CTLA-4: Cytotoxic T-lymphocyte-associated antigen 4
FBS: Fetal bovine serum
RIPA: Radioimmunoprecipitation assay
IHC: Immunohistochemical
BSA: Bovine serum albumin
HRP: Horseradish peroxidase
DAB: Diaminobenzidine
ROC: Receiver operating characteristic
ECM: Extracellular matrix
AUC: Area under the curve
CHOL: Cholangiocarcinoma
PAAD: Pancreatic adenocarcinoma
LIHC: Liver hepatocellular carcinoma
dMMR: Mismatch repair deficient
MDSCs: Myeloid-derived suppressor cells
TILs: Tumor infiltrating lymphocytes
VEGF: Vascular endothelial growth factor
ACVRL1: Activin-A receptor cluster II-like kinase 1
TGF: Transforming growth factor
